# The Role of Fortified and Enriched Refined Grains in the US Dietary Pattern: A NHANES 2009–2016 Modeling Analysis to Examine Nutrient Adequacy

**DOI:** 10.3389/fnut.2021.655464

**Published:** 2021-09-06

**Authors:** Yanni Papanikolaou, Victor L. III Fulgoni

**Affiliations:** ^1^Nutritional Strategies, Paris, ON, Canada; ^2^Nutrition Impact, Battle Creek, MI, United States

**Keywords:** NHANES, ready-to-eat breakfast cereals, breads, grains, shortfall nutrients

## Abstract

**Background:** While dietary recommendations call for greater whole-grain intake and reduced refined grain consumption, there are limited peer-reviewed studies examining the influence of fortified/enriched refined grains on nutrient adequacy.

**Methods:** A modeling analysis using data from National Health and Nutrition Examination Survey (NHANES) 2009–2016 estimated usual daily intake of shortfall nutrients for Dietary Guidelines for Americans (DGA) in the current dietary pattern and when specific percentages of fortified/enriched refined grain foods (bread, ready-to-eat cereals, and all-grained foods) were removed from the diet (19–50-year-old adults, *N* = 11,169; 51–99-year-old adults, *N* = 9,641).

**Results:** While American adults are currently falling short of nutrient recommendations, eliminating 25, 50, and 100% of all grains consumed in the US dietary pattern resulted in a greater percentage of adults not meeting recommendations for several shortfall nutrients, including dietary fiber, folate DFE, iron, and magnesium. Removal of all grains led to a reduced energy intake by ~10% in both age groups examined. Currently, ~3.8% of 19–50-year-old adults meet the adequate intake (AI) for dietary fiber. Removal of 25, 50, and 100% of grains from the diet resulted in 2.6 ± 0.3, 1.8 ± 0.2, and 0.7 ± 0.1% of adults exceeded the AI for dietary fiber, respectively. Similarly, 11.0 and 13.8% of younger and older adults, respectively, fall short of folate, DFE recommendations with the current diet. Following the removal of 100% of grains from the diet, 43.4 ± 1.1 and 56.2 ± 1.0%, respectively, were below the estimated average requirement (EAR) for folate DFE. For iron, current dietary pattern consumption shows 8.4% and 0.8% of younger and older adults, respectively, are not meeting iron recommendations, however, removal of 100% of grains from the diet results in nearly 10 and 22% falling short of the EAR. Currently, about 51 and 54% of younger and older adults are below the EAR for magnesium; however, with the removal of 100% of grains, 68 and 73%, respectively, fall below the EAR.

**Conclusion:** Removal of specific refined grains led to an increased percentage of Americans not meeting recommendations for several shortfall nutrients, including dietary fiber, folate, iron, and magnesium.

## Introduction

The 2020 Dietary Guidelines for Americans (2020 DGA) provide food and nutrition recommendations for all Americans of 2 years of age and older, with the promotion of dietary patterns emphasizing fruits, vegetables, whole grains, lean protein foods, and low-fat or fat-free dairy products, all of which are based on their contributing nutrient density to the American diet (1). While dietary guidance encourages increased consumption of whole grains, there are limitations placed on refined grain consumption (1). Both the 2015 DGA (2) and the 2020 DGA (1) have recommended that half of your daily total grain servings should be derived from whole grains. DGA (2015) further stated, “choosing both whole and refined grain foods in nutrient-dense forms, such as choosing plain popcorn instead of buttered, bread instead of croissants, and English muffins instead of biscuits also can help in meeting recommendations for a healthy eating pattern” (1). Accumulating observational evidence suggests that certain refined grain foods (i.e., ready-to-eat cereals, bread) may be important sources of nutrients, particularly, nutrients that have been identified as falling below recommended intakes in American populations (3–10). Shortfall nutrients have been defined as “nutrients that are under-consumed relative to requirement levels set by the Institute of Medicine (IOM) and includes dietary fiber, calcium, magnesium, potassium, vitamins A, C, D, and E, dietary folate, and iron in select female populations.” Dietary fiber, calcium, potassium, and vitamin D have been previously highlighted as “nutrients of public health concern” as chronic under consumption of these nutrients have been associated with unfavorable health conditions (1).

Recently published studies, that have assessed dietary sources of caloric energy and nutrients, suggest several grain-based foods contribute nutrient density to Americans of various age groups (3, 4, 7, 9, 10). Relative to caloric energy, certain grain-based foods delivered a substantial percentage of shortfall nutrients, including fiber, calcium, iron, magnesium, and folate. Grain-based foods, including breakfast cereals, bread, and tortillas have been shown to contribute ≥10% of fiber, iron, zinc, folate, niacin, and thiamin in the US dietary pattern (3, 4, 10). In addition, National Health and Nutrition Examination Survey (NHANES) findings have demonstrated that significant amounts of numerous essential nutrients originate from fortified and enriched foods, prompting researchers to conclude that documented nutrient shortfalls in Americans are further exacerbated without fortification and enrichment practices (11). Fortified and enriched foods have also been shown to positively contribute to the American diet. Specifically, fortification and enrichment practices in the US significantly contribute to iron, folate, thiamin, vitamin A, vitamin D, and vitamin C, and it has been suggested that without discretionary enrichment and/or fortification in the food supply, many Americans would not meet recommended nutrient intake levels (11).

Current US dietary recommendations promote greater intake of whole grains alongside the limitation of refined and/or enriched grain foods, however, many adults are not attaining recommended intakes for key nutrients, several of which originate from fortified and enriched grain foods. As grains represent a key food group in the US dietary pattern, it is hypothesized that specified grain foods (bread and ready-to-eat cereals) are associated with nutrient adequacy and can be an integral part of helping to meet dietary guidelines. The objective of the present study was to estimate usual intake for shortfall nutrients, in addition to enrichment and/or fortification nutrients, when 25–100% of specific grain-based foods are eliminated from the American diet.

## Methods

The present analysis used data from NHANES, thus providing a national, cross-sectional representation of free-living American adults of various age groups. NHANES data are compiled by the Centers for Disease Control and Prevention within the National Center for Health Statistics (NCHS). Written informed consent and prerequisite ethical protocols for all study participants are directed and granted by the NCHS Research Ethics Review Board. Four datasets (NHANES 2009–2010; 2011–2012; 2013–2014; 2015–2016) were used to complete the analyses in adults. Data representing nutrient intakes within NHANES 2009–2016 are derived from the US Department of Agriculture (USDA) Food and Nutrient Database for Dietary Studies (FNDDS), which contribute to the dietary nutrient values reported in What We Eat in America (WWEIA), the dietary intake section of NHANES (12–14). The WWEIA food categories provide an application to analyze food and beverages as consumed in the American diet. The classification scheme includes 150 unique categories and there are 15 main food groups and 46 subcategories of foods. WWEIA food categories have been previously published by USDA. Using WWEIA food categories and classifications, the present modeling analysis represented theoretical removal of bread, bread and cereals combined, and all enriched and/or fortified grain foods at 25, 50, and 100% elimination. The modeling analysis was meant to examine the nutrient contribution of grain foods (bread and ready-to-eat cereals and all grains) and potential impacts to achieving nutrient recommendations.

The NHANES dataset sample included 11,169 adults of 19–50-year-old and 9,641 adults of 51–99-year-old, where all subjects presented complete and reliable 24 h dietary intake recalls. Trained certified survey analysts administered dietary recalls *via* the Automated Multiple-Pass Method, a computerized, evidence-based procedure developed by USDA that encompasses a comprehensive description of foods and beverages and amounts consumed (15–17).

Two days of 24 h dietary recalls were used to determine usual intakes by using the methodology of the National Cancer Institute. Usual intake means, percentiles and percent meeting National Academy of Medicine Dietary Reference Intake (DRI) cutoffs [i.e., estimated average requirement (EAR) and adequate intake (AI)] were estimated using version 2.1 of the methodology of National Cancer Institute and were used to evaluate the impact of grain removal. The percentage of the population below the EAR or above the AI was assessed using the cut-point method, except for iron, which was assessed using the probability method (18). The methodology of National Cancer Institute (version 2.1) was used to estimate usual intake means, percentiles and percent meeting Dietary Reference Intake (DRI) cutoffs [i.e., EAR and AI] following the removal of specified percentages of grain foods from the typical US dietary patterns. The age groups, gender, day sequence, and weekend of DRI were covariates considered in the present analysis. The elimination and removal of grain models examined included the following: (a) “as is/no modeling changes” in dietary intake to represent typical and current scenarios of nutrient intake patterns; (b) 25, 50, and 100% removal of bread [WWEIA category 4202 (yeast bread)]; (c) 25, 50, and 100% removal of yeast bread, ready-to-eat cereals (RTEC) (WWEIA category 4202 or subgroup 46); and (d) 25, 50, and 100% elimination of grain-based foods [all-grained foods included in enrichment and fortification practices (i.e., classified as WWEIA main group 4)].

SAS software (Version 9.4, SAS Institute, Cary, NC, USA) was used to implement statistical analyses and SAS PROC SURVEYMEANS was used to determine means and percentages presented in the modeling scenarios. Survey weights used to generate representative estimates for U.S. adults also included survey weights to adjust for the complex sample design of NHANES.

## Results

### Energy Intake

Table 1 summarizes the mean usual energy intake in 19–50-year-old and 51–99-year-old adults. Removal of all bread and cereals leads to reductions of usual daily energy intake of 3–5% in younger adults and 4–6% in older adults. Removal of all grains led to a reduced energy intake by ~10% in both adult groups.

**Table 1:** Energy intakes: mean usual energy intakes in 19–50-year-old and 51–99-year-old adults.

			**Usual intake**	
	**Grain foods**	% Removal from current dietary pattern^*^	**Mean**	**SE**
**19–50 years-old**				
Energy (kcal)	Current	0	2,250	11.2
	Breads	25	2,215	11.4
	Breads	50	2,231	11.6
	Breads	100	2,215	11.4
	Breads + RTEC	25	2,223	11.6
	Breads + RTEC	50	2,194	10.1
	Breads + RTEC	100	2,141	10.9
	All Grains	25	2,185	11.0
	All Grains	50	2,118	11.2
	All Grains	100	1,987	11.4
**51–99 years-old**				
Energy (kcal)	Current	0	2013.0	10.0
	Breads	25	1922.0	10.0
	Breads	50	1968.0	10.3
	Breads	100	1925.0	7.8
	Breads + RTEC	25	1981.0	10.9
	Breads + RTEC	50	1947.0	9.3
	Breads + RTEC	100	1881.0	9.3
	All Grains	25	1945.0	9.7
	All Grains	50	1877.0	10.2
	All Grains	100	1743.0	9.8

*The data source is NHANES 2009–2016; N = 11,169; Usual intake determined using the National Cancer Institute method.*

* *Current dietary pattern using data collected from NHANES 2009–2016*.

### Nutrients of Public Health Concern

Tables 2, 3 summarize the mean usual nutrients of public health concern intakes and DRI percentages from 19- to 50-year-old and from 51 to 99-year-old adults, respectively.

**Table 2:** Nutrients of public health concern: mean usual nutrient intakes and percentages meeting recommendations in 19–50-year-old adults.

			**Usual intake**		** < EAR**		**>AI**	
**Nutrients**	**Grain foods**	% Removal from current dietary pattern^*^	**Mean**	**SE**	**%**	**SE**	**%**	**SE**
**Nutrients of Public Health Concern**								
Dietary fiber, g/d	Current	0	17.2	0.2			3.8	**0.4**
	Breads	25	17.0	0.2			3.5	0.3
	Breads	20	16.7	0.2			3.3	0.3
	Breads	100	16.2	0.2			2.7	0.3
	Breads+RTEC	25	16.8	0.2			3.1	0.3
	Breads+RTEC	50	16.3	0.2			2.5	0.3
	Breads+RTEC	100	15.4	0.2			1.7	0.2
	All Grains	25	16.4	0.2			2.6	0.3
	All Grains	50	15.5	0.2			1.8	0.2
	All Grains	100	13.9	0.2			0.7	0.1
Potassium, mg/d	Current	0	2,684	19.1			2.4	0.3
	Breads	25	2,678	18.1			2.3	0.2
	Breads	50	2,669	18.0			2.3	0.2
	Breads	100	2,646	18.6			2.1	0.3
	Breads+RTEC	25	2,667	18.8			2.3	0.2
	Breads+RTEC	50	2,648	18.0			2.1	0.2
	Breads+RTEC	100	2,614	17.4			1.9	0.2
	All Grains	25	2,648	18.5			2.1	0.2
	All Grains	50	2,609	17.6			1.9	0.2
	All Grains	100	2,534	18.2			1.4	0.2
Calcium, mg/d	Current	0	1,022	7.7	31.4	0.8		
	Breads	25	1,013	7.6	32.2	0.8		
	Breads	50	1,006	7.7	32.9	0.8		
	Breads	100	990	7.5	34.6	0.9		
	Breads+RTEC	25	1,008	7.8	32.6	0.8		
	Breads+RTEC	50	997	7.6	33.6	0.8		
	Breads+RTEC	100	974	7.6	36.0	0.9		
	All Grains	25	996	7.5	33.9	0.8		
	All Grains	50	969	7.5	36.8	0.8		
	All Grains	100	920	7.4	42.1	0.9		
Vitamin D^a^, μg/d	Current	0	4.6	0.08	95.4	0.4		
	Breads	25	4.6	0.08	95.4	0.4		
	Breads	50	4.6	0.08	95.6	0.4		
	Breads	100	4.6	0.08	95.4	0.4		
	Breads+RTEC	25	4.5	0.08	95.8	0.4		
	Breads+RTEC	50	4.5	0.07	96.2	0.4		
	Breads+RTEC	100	4.3	0.07	96.6	0.3		
	All Grains	25	4.5	0.07	95.8	0.4		
	All Grains	50	4.4	0.07	96.3	0.4		
	All Grains	100	4.2	0.07	96.8	0.3		

a *Vitamin D2+D3; EAR, Estimated Average Requirement; AI, Adequate Intake.*

*The data source is NHANES 2009–2016; N = 11,169; Usual intake determined using the National Cancer Institute method.*

* *Current dietary pattern using data collected from NHANES 2009–2016*.

**Table 3:** Nutrients of public health concern: mean usual nutrient intakes and percentages meeting recommendations in 51–99-year-old adults.

			**Usual intake**		** < EAR**		**>AI**	
**Nutrients**	**Grain foods**	% Removal from current dietary pattern^*^	**Mean**	**SE**	**%**	**SE**	**%**	**SE**
**Nutrients of Public Health Concern**
Dietary fiber, g/d	Current	0	17.6	0.2			14.1	0.7
	Breads	25	17.2	0.2			13.0	0.6
	Breads	50	16.9	0.2			11.9	0.7
	Breads	100	16.2	0.2			10.0	0.6
	Breads + RTEC	25	16.9	0.2			11.9	0.6
	Breads + RTEC	50	16.3	0.2			9.9	0.6
	Breads + RTEC	100	15.0	0.1			6.6	0.5
	All Grains	25	16.6	0.2			10.6	0.6
	All Grains	50	15.6	0.1			7.7	0.5
	All Grains	100	13.6	0.1			3.6	0.3
Potassium, mg/d	Current	0	2,750	16.4			2.6	0.3
	Breads	25	2,739	16.3			2.5	0.2
	Breads	50	2,723	17.4			2.4	0.3
	Breads	100	2,697	17.0			2.3	0.3
	Breads + RTEC	25	2,725	16.6			2.4	0.3
	Breads + RTEC	50	2,699	17.2			2.2	0.2
	Breads + RTEC	100	2,653	16.2			2.0	0.2
	All Grains	25	2,710	15.7			2.3	0.2
	All Grains	50	2,661	15.7			2.0	0.2
	All Grains	100	2,573	15.2			1.5	0.2
Calcium, mg/d	Current	0	931	8.4	56.0	1.0		
	Breads	25	920	8.5	57.2	1.0		
	Breads	50	910	8.6	58.2	1.0		
	Breads	100	889	8.6	60.2	1.0		
	Breads + RTEC	25	915	8.4	57.7	1.0		
	Breads + RTEC	50	901	8.3	59.2	1.0		
	Breads + RTEC	100	869	8.0	62.4	0.9		
	All Grains	25	902	8.2	59.1	0.9		
	All Grains	50	872	8.2	62.2	0.9		
	All Grains	100	812	7.8	68.2	0.9		
Vitamin D^a^, μg/d	Current	0	5.0	0.1	93.8	0.4		
	Breads	25	5.0	0.1	94.0	0.4		
	Breads	50	5.0	0.1	94.0	0.4		
	Breads	100	5.0	0.1	93.9	0.4		
	Breads + RTEC	25	4.9	0.1	94.5	0.4		
	Breads + RTEC	50	4.8	0.1	95.0	0.4		
	Breads + RTEC	100	4.6	0.1	95.6	0.4		
	All Grains	25	4.9	0.1	94.5	0.4		
	All Grains	50	4.8	0.1	95.2	0.4		
	All Grains	100	4.5	0.1	95.9	0.4		

a *Vitamin D2+D3; EAR, Estimated Average Requirement; AI, Adequate Intake.*

*The data source is NHANES 2009–2016; N = 9,641; usual intake determined using the National Cancer Institute method.*

* *Current dietary pattern using data from NHANES 2009–2016*.

Based on the current NHANES database, the percentage of individuals above the AI for dietary fiber is only 3.8 ± 0.4 and 14.1 ± 0.7%, in the younger and older adults, respectively. Removal of bread and the combination of removal of bread and RTEC further decreases the percentage of adults above the AI with the largest decline seen with the removal of all enriched and fortified grains from the diet, leaving only 0.7 ± 0.1 and 3.6 ± 0.3% of younger and older adults above the AI for dietary fiber, respectively. At present, 31.4 ± 0.8% of 19–50-year-old adults fall short of the EAR for calcium; however, the percentage increases to 34.6 ± 0.9, 36.0 ± 0.9, and 42.1 ± 0.9 when bread, bread and RTEC, and all grains are removed from the current dietary pattern, respectively. Similarly, for older adults (51–99 y) 56.0 ± 1.0% of adults fall short of the EAR for calcium; however, the percentage increases to 60.2 ± 1.0, 62.4 ± 0.9, and 68.2 ± 0.9% when bread, bread and RTEC, and all grains are removed from the current dietary pattern. Currently, only 2.4 ± 0.3 and 2.6 ± 0.3% are above the AI for potassium and 95.4 ± 0.4 and 93.8 ± 0.4% fall below the EAR for vitamin D, in younger and older adults, respectively, with minimal differences contributed from grain foods.

### Shortfall Nutrients

Tables 4, 5 summarize the mean usual shortfall nutrient intakes and DRI percentages from 19- to 50-year-old and from 51 to 99-year-old adults, respectively.

**Table 4:** Shortfall nutrients: mean usual nutrient intakes percentages meeting recommendations in 19–50-year-old adults.

			**Usual intake**		** < EAR**		**>AI**	
**Nutrients**	**Grain foods**	% Removal from current dietary pattern^*^	**Mean**	**SE**	**%**	**SE**	**%**	**SE**
**Shortfall Nutrients**								
Folate, μg DFE/d	Current	0	555	5.2	11.0	0.6		
	Breads	25	548	5.2	11.8	0.6		
	Breads	50	540	5.0	12.7	0.7		
	Breads	100	525	5.0	14.9	0.7		
	Breads+RTEC	25	525	4.6	12.4	0.7		
	Breads+RTEC	50	492	4.1	14.6	0.8		
	Breads+RTEC	100	428	3.6	24.5	0.9		
	All Grains	25	506	4.4	14.6	0.7		
	All Grains	50	457	3.9	20.1	0.8		
	All Grains	100	358	3.3	43.4	1.1		
Iron, mg/d	Current	0	15.2	0.1	8.4	0.3		
	Breads	25	15.1	0.1	8.9	0.3		
	Breads	50	14.8	0.1	9.3	0.3		
	Breads	100	14.5	0.1	10.3	0.4		
	Breads+RTEC	25	14.5	0.1	9.4	0.3		
	Breads+RTEC	50	13.7	0.1	10.7	0.4		
	Breads+RTEC	100	12.3	0.1	14.8	0.4		
	All Grains	25	14.1	0.1	10.4	0.3		
	All Grains	50	12.9	0.1	12.9	0.4		
	All Grains	100	10.5	0.1	21.9	0.5		
Magnesium, mg/d	Current	0	312	2.4	50.8	1.0		
	Breads	25	309	2.4	52.0	1.0		
	Breads	50	306	2.4	53.1	1.0		
	Breads	100	301	2.3	55.1	1.0		
	Breads+RTEC	25	307	2.4	52.8	1.0		
	Breads+RTEC	50	302	2.3	54.9	1.0		
	Breads+RTEC	100	293	2.3	58.9	1.0		
	All Grains	25	302	2.3	55.0	1.0		
	All Grains	50	292	2.2	59.2	1.0		
	All Grains	100	273	2.2	67.6	1.0		
Vitamin A, μg RAE/d	Current	0	614	8.6	49.2	1.2		
	Breads	25	614	9.2	49.0	1.2		
	Breads	50	613	8.8	49.2	1.2		
	Breads	100	613	8.7	49.3	1.2		
	Breads+RTEC	25	601	8.5	51.0	1.2		
	Breads+RTEC	50	588	8.4	52.9	1.2		
	Breads+RTEC	100	563	8.1	56.8	1.2		
	All Grains	25	596	8.5	51.6	1.2		
	All Grains	50	577	8.1	54.5	1.2		
	All Grains	100	541	7.8	60.3	1.2		
Vitamin C, mg/d	Current	0	79.4	1.3	48.9	1.1		
	Breads	25	79.5	1.3	48.9	1.1		
	Breads	50	79.5	1.3	48.9	1.1		
	Breads	100	79.5	1.3	48.9	1.1		
	Breads+RTEC	25	79.0	1.3	49.4	1.1		
	Breads+RTEC	50	78.5	1.3	49.9	1.1		
	Breads+RTEC	100	77.8	1.3	50.7	1.1		
	All Grains	25	79.0	1.3	49.5	1.1		
	All Grains	50	78.5	1.3	49.9	1.1		
	All Grains	100	77.7	1.3	50.8	1.1		
Vitamin E^a^, mg/d	Current	0	9.0	0.1	81.3	0.8		
	Breads	25	8.9	0.1	81.6	0.8		
	Breads	50	8.9	0.1	81.6	0.8		
	Breads	100	8.8	0.1	82.0	0.8		
	Breads+RTEC	25	8.9	0.1	82.3	0.8		
	Breads+RTEC	50	8.8	0.1	82.9	0.7		
	Breads+RTEC	100	8.5	0.1	84.5	0.7		
	All Grains	25	8.8	0.1	82.6	0.8		
	All Grains	50	8.6	0.1	83.8	0.8		
	All Grains	100	8.3	0.1	86.0	0.7		

a *as alpha-tocopherol; EAR, Estimated Average Requirement; AI, Adequate Intake.*

*The Data source is NHANES 2009–2016; N = 9,641; Usual intake determined using the National Cancer Institute method.*

* *Current dietary pattern using data collected from NHANES 2009–2016*.

**Table 5:** Shortfall nutrients: mean usual nutrient intakes, usual intake percentiles, and DRI percentages in 51–99-year-old adults.

			**Usual intake**		** < EAR**		**>AI**	
**Nutrients**	**Grain foods**	% Removal from current dietary pattern^*^	**Mean**	**SE**	**%**	**SE**	**%**	**SE**
**Shortfall Nutrients**								
Folate, μg DFE/d	Current	0	523	4.4	13.8	0.8		
	Breads	25	513	4.4	15.2	0.9		
	Breads	50	503	4.3	16.6	0.9		
	Breads	100	483	4.1	20.0	0.9		
	Breads + RTEC	25	487	3.8	16.7	0.9		
	Breads + RTEC	50	450	3.5	20.8	0.9		
	Breads + RTEC	100	378	3.2	37.0	1.0		
	All Grains	25	472	3.8	19.0	0.9		
	All Grains	50	420	3.3	27.0	1.0		
	All Grains	100	319	2.8	56.2	1.0		
Iron, mg/d	Current	0	14.6	0.1	0.8	0.1		
	Breads	25	14.3	0.1	0.9	0.1		
	Breads	50	14.1	0.1	1.1	0.2		
	Breads	100	13.5	0.1	1.7	0.2		
	Breads + RTEC	25	13.7	0.1	1.0	0.2		
	Breads + RTEC	50	12.8	0.1	1.4	0.2		
	Breads + RTEC	100	11.0	0.1	4.3	0.3		
	All Grains	25	13.3	0.1	1.4	0.2		
	All Grains	50	12.0	0.1	2.2	0.2		
	All Grains	100	9.4	0.1	9.9	0.5		
Magnesium, mg/d	Current	0	304	2.1	54.2	0.9		
	Breads	25	301	2.0	55.7	0.9		
	Breads	50	297	2.1	57.3	0.9		
	Breads	100	290	2.0	60.3	0.8		
	Breads + RTEC	25	298	2.1	56.9	0.9		
	Breads + RTEC	50	292	2.0	59.5	0.9		
	Breads + RTEC	100	279	2.1	65.1	0.9		
	All Grains	25	293	2.0	59.0	0.8		
	All Grains	50	282	2.0	63.9	0.9		
	All Grains	100	260	1.8	73.0	0.9		
Vitamin A, μg RAE/d	Current	0	682	8.9	38.6	1.1		
	Breads	25	682	9.4	38.4	1.1		
	Breads	50	682	8.7	38.6	1.1		
	Breads	100	682	8.9	38.6	1.1		
	Breads + RTEC	25	667	8.7	40.3	1.1		
	Breads + RTEC	50	651	8.3	42.3	1.0		
	Breads + RTEC	100	622	7.6	46.6	1.0		
	All Grains	25	659	8.5	41.2	1.1		
	All Grains	50	639	8.4	44.0	1.1		
	All Grains	100	594	7.6	50.8	1.1		
Vitamin C, mg/d	Current	0	87.0	1.5	41.9	1.1		
	Breads	25	86.9	1.4	41.9	1.0		
	Breads	50	87.1	1.6	41.9	1.1		
	Breads	100	86.9	1.6	42.0	1.1		
	Breads + RTEC	25	86.4	1.5	42.5	1.1		
	Breads + RTEC	50	85.9	1.6	42.9	1.1		
	Breads + RTEC	100	84.8	1.6	44.1	1.1		
	All Grains	25	86.5	1.6	42.4	1.1		
	All Grains	50	85.9	1.6	43.0	1.1		
	All Grains	100	84.6	1.6	44.2	1.1		
Vitamin E^a^, mg/d	Current	0	8.8	0.1	82.6	0.9		
	Breads	25	8.8	0.1	82.9	0.9		
	Breads	50	8.7	0.1	83.1	0.9		
	Breads	100	8.7	0.1	83.7	0.9		
	Breads + RTEC	25	8.7	0.1	83.7	0.8		
	Breads + RTEC	50	8.5	0.1	84.7	0.9		
	Breads + RTEC	100	8.2	0.1	86.8	0.7		
	All Grains	25	8.6	0.1	84.2	0.8		
	All Grains	50	8.4	0.1	85.5	0.8		
	All Grains	100	8.0	0.1	88.0	0.7		

a *as alpha-tocopherol; EAR, Estimated Average Requirement; AI, Adequate Intake.*

*The data source is NHANES 2009–2016; N = 9,641; Usual intake determined using the National Cancer Institute method.*

* *Current dietary pattern using data from NHANES 2009–2016*.

The current analysis further substantiates that fortification and enrichment help contribute to the achievement of nutrient adequacy in the American diet. At present, from 19- to 50-year-old and from 51- to 99-year-old adults, 11.0 ± 0.6 and 13.8 ± 0.8% fall below the EAR for folate, while 8.4 ± 0.3 and 0.8 ± 0.1% are below the EAR for iron, respectively. Removal of bread, bread and RTEC, and all grains result in meaningful increases in the percentage of individuals below the EAR in all adults. Indeed, while many Americans are not meeting recommendations for magnesium, vitamin A, vitamin C, and vitamin E, the removal of bread, bread and RTEC and all grains further exacerbates the percentage of adults not meeting the EAR for these nutrients.

### Other Discretionary Enrichment Nutrients

Tables 6, 7 summarize mean usual riboflavin, thiamin and niacin intakes, and DRI percentages from 19- to 50-year-old and from 51- to 99-year-old adults, respectively. Due to current enrichment practices, a small percentage of adults fall below the EAR for riboflavin, thiamin and niacin in both age groups examined. Grain foods are likely to play an important role in helping to achieve nutrient adequacy in the American adult population as evidenced by modeling the removal of grains from the diet. Indeed, the removal of all enriched and fortified grains from the diet in the age group from 19- to 50-year-old and from 51- to 99-year-old adults increased the percent of adults falling beneath the thiamin EAR, i.e., from 5.6 ± 0.4 to 28.2 ± 1.0% and from 7.8 ± 0.6 to 39.2 ± 0.9%, respectively. Similarly, the removal of all enriched and fortified grains from the diet in the age group of 19–50- and 51–99-year-old adults elevated the percent of adults not attaining the EAR for niacin, adults below the niacin EAR increased from 0.8 ± 0.1 to 4.3 ± 0.4% and from 2.0 ± 0.2 to 11.0 ± 0.7%, respectively.

**Table 6:** Discretionary enrichment nutrients: mean usual nutrient intakes and percentages meeting recommendations in 19–50-year-old adults.

			**Usual intake**		** < EAR**		**>AI**	
**Nutrients**	**Grain foods**	% Removal from current dietary pattern^*^	**Mean**	**SE**	**%**	**SE**	**%**	**SE**
**Discretionary Enrichment Nutrients**								
Riboflavin, mg/d	Current	0	2.18	0.02	3.01	0.24		
	Breads	25	2.16	0.02	3.21	0.25		
	Breads	50	2.14	0.02	3.44	0.25		
	Breads	100	2.11	0.02	3.93	0.25		
	Breads+RTEC	25	2.12	0.02	3.31	0.28		
	Breads+RTEC	50	2.07	0.02	3.72	0.28		
	Breads+RTEC	100	1.97	0.02	5.05	0.32		
	All Grains	25	2.09	0.02	3.69	0.27		
	All Grains	50	2.01	0.02	4.50	0.31		
	All Grains	100	1.85	0.02	7.42	0.45		
Thiamin, mg/d	Current	0	1.68	0.01	5.58	0.43		
	Breads	25	1.65	0.01	6.19	0.47		
	Breads	50	1.62	0.01	6.88	0.49		
	Breads	100	1.57	0.01	8.81	0.56		
	Breads+RTEC	25	1.61	0.01	6.51	0.49		
	Breads+RTEC	50	1.55	0.01	7.85	0.57		
	Breads+RTEC	100	1.43	0.01	12.94	0.74		
	All Grains	25	1.56	0.01	7.99	0.55		
	All Grains	50	1.44	0.01	11.99	0.68		
	All Grains	100	1.21	0.01	28.16	0.97		
Niacin, mg/d	Current	0	27.72	0.20	0.76	0.10		
	Breads	25	27.42	0.20	0.85	0.11		
	Breads	50	27.15	0.20	0.93	0.13		
	Breads	100	26.56	0.20	1.24	0.16		
	Breads+RTEC	25	26.96	0.19	0.89	0.12		
	Breads+RTEC	50	26.21	0.19	1.10	0.15		
	Breads+RTEC	100	24.69	0.18	2.13	0.26		
	All Grains	25	26.48	0.19	1.08	0.14		
	All Grains	50	25.26	0.19	1.60	0.19		
	All Grains	100	22.82	0.19	4.25	0.40		

*EAR, Estimated Average Requirement; AI, Adequate Intake.*

*The data source is NHANES 2009–2016; N = 9,641; Usual intake determined using the National Cancer Institute method.*

* *Current dietary pattern using data from NHANES 2009-2016*.

**Table 7:** Discretionary enrichment nutrients: mean usual nutrient intakes, usual intake percentiles, and DRI, and percentages meeting recommendations in 51–99-year-old adults.

			**Usual intake**		** < EAR**		**>AI**	
**Nutrients**	**Grain foods**	% Removal from current dietary pattern^*^	**Mean**	**SE**	**%**	**SE**	**%**	**SE**
**Discretionary Enrichment Nutrients**								
Riboflavin, mg/d	Current	0	2.2	0.01	2.8	0.2		
	Breads	25	2.1	0.01	3.0	0.2		
	Breads	50	2.1	0.01	3.2	0.2		
	Breads	100	2.1	0.01	3.8	0.2		
	Breads + RTEC	25	2.1	0.01	3.1	0.2		
	Breads + RTEC	50	2.0	0.01	3.6	0.2		
	Breads + RTEC	100	1.9	0.01	5.2	0.3		
	All Grains	25	2.1	0.01	3.5	0.2		
	All Grains	50	2.0	0.01	4.4	0.3		
	All Grains	100	1.8	0.01	7.8	0.4		
Thiamin, mg/d	Current	0	1.6	0.01	7.8	0.6		
	Breads	25	1.5	0.01	8.8	0.6		
	Breads	50	1.5	0.01	10.1	0.7		
	Breads	100	1.4	0.01	13.5	0.7		
	Breads + RTEC	25	1.5	0.01	9.5	0.7		
	Breads + RTEC	50	1.4	0.01	12.1	0.7		
	Breads + RTEC	100	1.3	0.01	21.1	0.8		
	All Grains	25	1.5	0.01	11.5	0.7		
	All Grains	50	1.3	0.01	17.5	0.8		
	All Grains	100	1.1	0.01	39.2	0.9		
Niacin, mg/d	Current	0	24.4	0.2	2.0	0.2		
	Breads	25	24.0	0.2	2.2	0.3		
	Breads	50	23.6	0.2	2.6	0.3		
	Breads	100	22.8	0.2	3.6	0.4		
	Breads + RTEC	25	23.5	0.2	2.5	0.3		
	Breads + RTEC	50	22.6	0.2	3.2	0.3		
	Breads + RTEC	100	20.9	0.2	6.4	0.5		
	All Grains	25	23.0	0.2	2.9	0.3		
	All Grains	50	21.8	0.2	4.3	0.4		
	All Grains	100	19.2	0.2	10.8	0.7		

*EAR, Estimated Average Requirement; AI, Adequate Intake. *

*The data source is NHANES 2009–2016; N = 9,641; usual intake determined using the National Cancer Institute method.*

* *Current dietary pattern using data from NHANES 2009–2016*.

## Discussion

Based on published peer-reviewed literature and to the best of our knowledge, no studies have been completed examining the potential nutrient intake consequences resulting from the removal and/or elimination of specific grain foods from the American diet, particularly, in the presence of mandated fortification and enrichment policy guidelines in the US. Indeed, prior to conducting the present grain-based foods analysis, current dietary patterns in American adults still reveal a meaningful percentage of adults are falling below recommendations for several nutrients, including vitamin A, vitamin C, vitamin D, vitamin E, magnesium, fiber, and dietary folate. The removal of bread and ready-to-eat cereals from the diet results in more adults falling short of recommendations for several nutrients, thus, further exacerbating nutrient inadequacy in Americans. Similarly, removing or reducing all grains (both whole and enriched grains) from the diet results in higher percentages of adults not meeting recommended intake levels for key nutrients, including several shortfall nutrients as distinguished by present and previous DGA. The current analysis substantiates the continued inclusion of whole and enriched grain foods as a part of the American adult eating pattern. The current analysis also illustrates the nutritional value of grain enrichment and fortification practices in the US, as a dietary strategy to help close nutrient recommendation gaps.

Since whole grains are typically not included in government-mandated fortification and enrichment practices in the US (19), there may be a misconception that refined and/or enriched grains are not required in the American diet. Indeed, while the 2015 DGAC recommended making half of total grain intake as whole grain, the inclusion of refined grains in the diet was supported by modeling studies that demonstrated the nutrient contribution of fortified and enriched grains (2). Furthermore, the perception that all refined grains are negative dietary components may lead to unintended nutrient intake and public health consequences. Indeed, the classification of all grains into the classification of refined grains when criteria for whole grain are not satisfied may be nutritionally flawed since many enriched grains (i.e., breakfast cereals, bread, cooked cereals, etc.) are nutrient-dense. Previous reports suggest <5% of the US population meets the minimum recommendation for whole-grain consumption, with the average American adult eating <1 oz equivalent of whole grains daily (1, 20). Therefore, it is a reasonable assumption that refined and/or enriched grains are the principal types of grains eaten by American consumers. Indeed, studies from this group using NHANES show that certain grain foods are key contributors of under-consumed nutrients (3, 4, 10). Analyses from earlier NHANES datasets in US children and adults have also corroborated with the current findings by highlighting enriched and/or fortified foods as key contributors of iron, folate, niacin, thiamin, riboflavin, vitamins A, vitamin B6, vitamin B12, vitamin C, and vitamin D. Indeed, the percentage of the population with usual intakes less than the EAR for many nutrients was significantly greater when only naturally occurring nutrients were considered in the diet. Enriched and/or fortified foods contributed to significant amounts of nutrients, such that the percentage of the population with usual intakes below the EAR significantly decreased for iron (22–7%), folate (88–11%), vitamin A (74–45%), and thiamin (51–6%) (11). The researchers concluded that nutrient intake shortfalls in Americans would further worsen following the removal of enriched and/or fortified foods (11).

While previous studies have focused on children and all adults (≥19-year-old) with no current data isolating older Americans (i.e., >50-year-old), the current study examined both younger adults (19–50-year-old) and older adults (51–99-year-old). Older Americans represent a substantial proportion of the US population with the CDC estimating that those ≥ 65-year-old will increase by 100% in the next 2.5 decades to nearly 72 million (21). Healthy aging is a multivariable process, with a key determinant of success involving meeting recommended nutrient intakes and routine consumption of a healthy dietary pattern, to optimize health and quality of life (22). Thus, intake of both macro- and micro-nutrients are critical factors in healthy aging. While it has been established that some grain-based food products provide added sugar, sodium, and saturated fat, several grain-based foods contribute numerous nutrients to encourage in older adults, including calcium, magnesium, dietary fiber, riboflavin, niacin, folate, and vitamin B12] (1, 10, 23, 24). Dietary fiber remains a shortfall nutrient in older Americans, with data showing about 4% of males and 13% of females consuming fiber above recommended levels (2). In addition, calcium intakes remain below recommendation with previous dietary guidance policy suggesting that 71% of males and 81% of females ≥71-year-old having daily intakes below the EAR. Magnesium and folate and are also under-consumed compared with the EAR in Americans (2). Previously published NHANES data further show that the percent of adults below the EAR gradually increases with age for several nutrients, including calcium, magnesium, folate, vitamin D, and vitamin E (25). Importantly, the current modeling data show that specific grain foods are important contributors of nutrients in the diet. Removal or elimination of grains in older adults resulted in meaningful increases in the percent of individuals not meeting recommendations for several shortfall nutrients, including dietary fiber, calcium, folate, iron, magnesium, vitamin A, thiamin, niacin, and riboflavin.

Study limitations inherent in the present study are typical of observational research and have been routinely acknowledged in preceding publications (3–6, 10). Dietary recall data require trust in the memory function of study participants, and as such, recalled information may include a level of errors, inaccuracies, and/or personal bias which are often present in sizeable data sets (26); however, the critical advantage of NHANES encompasses the capacity to direct research analyses grounded in a large and nationally representative dataset of American adults with associations to food and beverage consumption and individualized nutrient intakes (14, 17). The current analysis only considered the percentage removal and/or complete elimination of specific fortified and enriched refined grain foods from the American diet. As such, future research can incorporate a modeling scenario that identifies results from replacing grain foods with other food group options to compare and contrast the nutritional contributions from grains relative to other food choices. While refined grain foods can be significant contributors of unfavorable nutrients in the diet, it is imperative to highlight that the current simulation did not include modeling for reductions in total sugars, added sugars, sodium, saturated fat, and other nutrients following the removal of grain foods from the dietary pattern. Future research by the group will consider these aspects in upcoming modeling that will also include substitutions to compliment the removal of foods in various age groups, including children and adults.

In conclusion, the current dietary pattern in American adults shows many adults are falling below recommendation levels for several nutrients, including dietary fiber, magnesium, folate, vitamin A, vitamin C, vitamin D, and vitamin E. The removal of bread and ready-to-eat cereals from the diet resulted in a higher percentage of adults falling short of recommended nutrient intakes. Similarly, removing or eliminating all grains from the diet further exacerbates those falling short of nutrient recommendations, leading to insufficient intake of several 2015–2020 DGA shortfall nutrients. The current analysis also illustrates the nutritional value of grain enrichment and fortification practices in the US, as a dietary strategy to help close nutrient recommendation gaps. In addition, while many Americans meet recommendations for several of the nutrients examined, including thiamin, riboflavin, and niacin, likely due in large part to mandated enrichment practices of grain foods in the US diet, removal and/or elimination of grains results in a greater percentage of the population falling below the respective EARs. Recommendations that call for the lowering of certain fortified and/or enriched grain foods from the American diet may be misaligned with public health initiatives aiming to improve nutrient adequacy in the American adult population. Rather, dietary recommendations should focus on balancing the consumption of nutrient-dense fortified/enriched refined and whole-grain foods.

## Data Availability Statement

Publicly available datasets were analyzed in this study. This data can be found here: https://www.cdc.gov/nchs/nhanes/index.htm.

## Ethics Statement

The studies involving human participants were reviewed and approved by Centers for Disease Control and Prevention. The patients/participants provided their written informed consent to participate in this study.

## Author Contributions

YP and VF routinely partner on research study design, conception, data interpretation, publication of NHANES results, and interpretation of the research. VF completed all analyses of the final study. YP drafted the original manuscript. Both authors collaborated on drafting the final manuscript and approval of the submitted version.

## Conflict of Interest

The authors declare that the research was conducted in the absence of any commercial or financial relationships that could be construed as a potential conflict of interest.

## Publisher's Note

All claims expressed in this article are solely those of the authors and do not necessarily represent those of their affiliated organizations, or those of the publisher, the editors and the reviewers. Any product that may be evaluated in this article, or claim that may be made by its manufacturer, is not guaranteed or endorsed by the publisher.

## Abbreviations

DGA: Dietary Guidelines for Americans
WWEIA: What We Eat in America
NHANES: National Health and Nutrition Examination Survey
USDA: United States Department of Agriculture
EAR: Estimated Average Intake
AI: Adequate Intake.
